# Spectrum Slicing for Multiple Access Channels with Heterogeneous Services

**DOI:** 10.3390/e23060686

**Published:** 2021-05-28

**Authors:** Federico Chiariotti, Israel Leyva-Mayorga, Čedomir Stefanović, Anders E. Kalør, Petar Popovski

**Affiliations:** Department of Electronic Systems, Aalborg University, Fredrik Bajers Vej 7C, 9100 Aalborg, Denmark; ilm@es.aau.dk (I.L.-M.); cs@es.aau.dk (Č.S.); aek@es.aau.dk (A.E.K.); petarp@es.aau.dk (P.P.)

**Keywords:** Age of Information, Non-Orthogonal Multiple Access, reliability, heterogeneous access, slotted ALOHA

## Abstract

Wireless mobile networks from the fifth generation (5G) and beyond serve as platforms for flexible support of heterogeneous traffic types with diverse performance requirements. In particular, the broadband services aim for the traditional rate optimization, while the time-sensitive services aim for the optimization of latency and reliability, and some novel metrics such as Age of Information (AoI). In such settings, the key question is the one of spectrum slicing: how these services share the same chunk of available spectrum while meeting the heterogeneous requirements. In this work we investigated the two canonical frameworks for spectrum sharing, Orthogonal Multiple Access (OMA) and Non-Orthogonal Multiple Access (NOMA), in a simple, but insightful setup with a single time-slotted shared frequency channel, involving one broadband user, aiming to maximize throughput and using packet-level coding to protect its transmissions from noise and interference, and several intermittent users, aiming to either to improve their latency-reliability performance or to minimize their AoI. We analytically assessed the performances of Time Division Multiple Access (TDMA) and ALOHA-based schemes in both OMA and NOMA frameworks by deriving their Pareto regions and the corresponding optimal values of their parameters. Our results show that NOMA can outperform traditional OMA in latency-reliability oriented systems in most conditions, but OMA performs slightly better in age-oriented systems.

## 1. Introduction

The fifth generation of mobile networks (5G) was designed to support three main types of services with widely different requirements: enhanced mobile broadband (eMBB), ultra-reliable low-latency communications (URLLC), and massive machine-type communications (mMTC) [1]. The eMBB category focuses on human-oriented services that transmit large amounts of data and offer higher data rates and increased spectral efficiency when compared to the previous generation. On the other hand, Internet of Things (IoT)-like services, which transmit small amounts of data intermittently (and hence are termed intermittent services throughout the rest of the paper), may fall within either URLLC or mMTC categories, depending on their latency and reliability requirements, and processing/computational capabilities. The intermittent services where low latency (in the order of a few milliseconds) must be guaranteed with extremely high reliability (in the order of 1–$10^{-5}$) belong to URLLC service type. Conversely, intermittent services with relaxed latency and reliability requirements while incorporating exceedingly large numbers of devices belong to mMTC service type.

However, such a categorization of IoT services is too simplistic and cannot model a finer gradation of timely data delivery requirements. In particular, there are novel, timeliness-related metrics that may better capture the requirements of some categories of IoT applications. In this respect, AoI has recently attracted attention due its ability to measure the freshness of information by combining the communication and data generation processes [2]. AoI is measured at the point of reception, as the time elapsed since the moment of generation (at the transmitter) of the last successfully received message. A related metric is Peak Age of Information (PAoI), which represents the AoI measured immediately before a new message is successfully received [3].

AoI and PAoI are particularly relevant in control systems and similar setups with (quasi) periodic message exchanges [4]. The underlying assumption is that users send updates of an ongoing process, such that the most recent update provides all the necessary information about the state of the process. In such scenario, the reliability and latency of individual packets are of secondary importance [2]. We refer the interested reader to a recent survey [5] for a thorough review of AoI and its properties, and to our previous work for a discussion on the differences between AoI and latency and reliability as timeliness metrics [6,7].

The concept of network slicing has been widely investigated in recent years, mainly motivated by the need for accommodation of heterogeneous services in the network. The idea is to allocate (i.e., slice) the network’s resources among the different coexisting services, such that each service has the experience of meeting the performance requirements while being isolated from the other service types [8,9]. In our previous work [7], we introduced the concept of spectrum slicing to refer to the allocation of shared wireless resources among coexisting heterogeneous services in the Radio Access Network (RAN). Those resources can be defined, for example, in time, frequency, or spatial domains. So far, spectrum allocation, rather than slicing, has been widely studied in the form of diverse OMA and NOMA techniques in the presence of multiple users with the same type of service [10,11,12]. OMA techniques assign dedicated resources to individual users and/or services: Orthogonal Frequency-Division Multiple Access (OFDMA), Code Division Multiple Access (CDMA), and multi-user multiple-input multiple-output (MU-MIMO) are examples of OMA that achieved widespread implementation in 3GPP cellular systems, including 5G [13,14,15]. On the other hand, the NOMA concept refers to the allocation of shared (i.e., non-orthogonal) resources in the time and/or frequency domains to multiple services or users. Such allocation intrinsically implies collisions of users’ transmissions in the shared domain(s), and NOMA techniques generally rely on more complex receivers, capable of Multi-Packet Reception (MPR) to resolve collisions. The benefit of this techniques is potentially a higher resource efficiency than OMA, and less need for strict coordination among users. On the downside, implementation of MPR techniques is usually complex; a typical example is Successive Interference Cancellation (SIC) [11,16].

**Figure 1:**
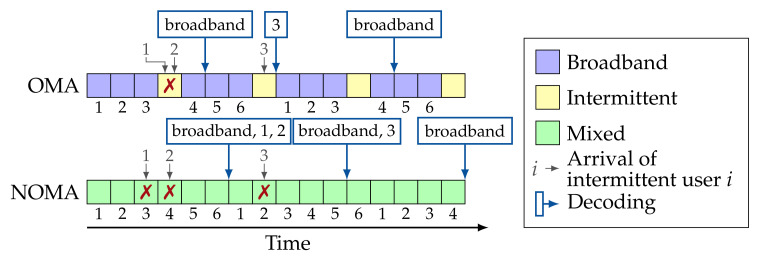
Toy example for for a case where the broadband user implements a 4-out-of-6 erasure code. No channel erasures are considered in this example. Collisions among intermittent users cannot be recovered, but SIC can be used to recover collisions between the broadband and intermittent users after decoding the broadband user.

In scenarios with broadband services only, resource efficiency is easily defined and the trade-offs are clearly characterized by the achievable data rates and/or throughput [11,12]. However, further research is needed on novel slicing mechanisms in scenarios with heterogeneous services, for example, broadband and intermittent, since the efficiency cannot be simply measured in terms of throughput or data rates [9,17]. We illustrate this through a toy example presented in Figure 1, where there are (i) 3 intermittent users following an ALOHA-based protocol, and (ii) a broadband user. With OMA, orthogonal resources are defined for each service type. This limits the frequency of resources for the intermittent users, which increases the probability of collision among them, as shown by the cross-mark in Figure 1; these collided packets cannot be recovered. In contrast, with NOMA all resources are available for the intermittent and broadband users, and SIC is used to recover the packets lost due to collision between the broadband and intermittent users. In this example, NOMA obtains greater throughout for the broadband user and a lower latency and greater reliability for the intermittent users than OMA. This insight motivates the work presented in this paper.

In particular, in this paper we investigate orthogonal and non-orthogonal slicing mechanisms in the case where a broadband user shares a wireless channel with multiple intermittent users share. Specifically, we explore the performance of slicing implemented via multiple access schemes standardly used in the cellular access, which are TDMA and slotted ALOHA, and a scheme representing their combination. The broadband user implements a *K*-out-of-*N* erasure code, which allows the user to counteract the packet losses due to channel and potential collisions with the intermittent users transmission in the case of non-orthogonal slicing. In the later case, once the block of *N* broadband users’ packets becomes decoded, the receiver uses SIC to attempt recovery of the intermittent users’ packets. The performance parameters of interests are throughput of the broadband user and two timeliness metrics for the intermittent users: latency-reliability of individual packets and PAoI.

In our previous works [6,7], we investigated the performance trade-offs of OMA and NOMA in a simple uplink scenario with one broadband user and one intermittent user. The general conclusion was that OMA usually outperforms NOMA when transmissions takes place in a collision channel with packet erasures and without capture, which is a rather conservative channel model. However, NOMA schemes achieved a similar performance as OMA in extreme cases when the single objective is to maximize the throughput of the broadband user or to minimize the latency of the intermittent user [6]. We also evaluated how the capture effect and immediate (i.e., intra-collision) SIC at the receiver enhance the performance of NOMA. Under this scenario we observed that important gains can be achieved with NOMA when the intermittent user aims to minimize latency, but the gains are limited when the objective is to minimize AoI [7]. This paper extends that analysis to the case with multiple intermittent users, showing quite different trade-offs. We derive closed-form expressions for the performance parameters and show that, when the intermittent users aim to minimize the PAoI, OMA with TDMA is the best choice, albeit by a small margin. In contrast, when the intermittent users aim to optimize the packet latency, the slicing mechanism must be carefully selected based on the access load and the number of users, as there is no single slicing method that provides the best trade-offs.

In summary, the main contributions of this paper are the following:

- We analyze the trade-offs and regions of operation of OMA and NOMA schemes with a broadband and multiple intermittent users in a collision channel with erasures.
- We investigate the impact of the metrics of interest on the overall system design and on the achievable gains with OMA and NOMA.
- We investigate the impact of the activation probability of intermittent users on the performance of the slicing mechanism.
- We derive Pareto frontiers, which define the best possible trade-offs between throughput of the broadband user and latency/AoI of the intermittent users with the considered schemes.

The rest of the paper is organized as follows. Section 2 presents the literature review. The system model is described in Section 3. The analyses for OMA and NOMA schemes are presented in Section 4 and Section 5, respectively. The results are presented in Section 6. Section 7 concludes the paper.

## 2. Related Work

Orthogonal slicing has been widely explored and used in commercial systems [9]. It is a straightforward approach where independent resources are allocated to the different services, which allows one to treat them in an isolated manner. Popovski et al. [17] provided one of the first studies that compared orthogonal to non-orthogonal slicing. In particular, it investigated the benefits of OMA and NOMA schemes for the different combinations of 5G services in an uplink scenario: eMBB with URLLC and eMBB with mMTC. In the latter, orthogonal resources were allocated to each eMBB user, mMTC traffic was assumed to be Poisson distributed, and one URLLC user was considered. It was observed that NOMA may offer benefits with respect to OMA depending on the rate of the eMBB users and on the coexisting type of the intermittent traffic: with high URLLC, high data rates at the eMBB user were beneficial for NOMA, whereas the opposite is true with mMTC traffic.

The work presented in [17] was extended to a multi-cell scenario with strict latency guarantees for URLLC traffic [18]. A single URLLC user per cell was considered, and it was observed that NOMA leads to a greater spectral efficiency with respect to OMA. A similar conclusion was drawn by Maatouk et al. [10] in an uplink scenario with two users with the same service type that aimed to minimize the average AoI. It was also observed that a greater spectral efficiency does not directly translate into a lower average AoI. Another scenario that includes power control to simplify the reception of the intermittent packets was studied in [19], which derived analytical formulas for throughput and AoI with those settings.

The selection of the multiple access scheme is essential when considering spectrum slicing with multiple intermittent users [9], and particularly so in MU-MIMO systems which can make MPR easier [20]. Slotted ALOHA and TDMA are two basic multiple access schemes that offer widely different benefits. Slotted ALOHA is simple, flexible, and effective for relatively low traffic loads. It is one of the most widely used random access protocols, implemented in a number of variants, e.g., multichannel slotted ALOHA in 5G [21,22]. There is a vast literature on the performance evaluation of ALOHA-based schemes in terms of latency and reliability. For instance, grant-free ALOHA-based access has been studied for URLLC services [23,24]. Besides, latency and reliability can be combined into a single performance indicator termed latency-reliability [25]. On the other hand, it is difficult to derive closed-form expressions of the probability distribution of the AoI. Hence, most papers in the literature examined it in terms of its mean value and in the context of queuing theory and often in ideal systems with Markovian service [26]. Only a few studies investigated the tail or the full distribution of AoI, event though these provide a clear measure for the reliability and stability of control systems. In particular, these are directly connected to control systems by the survival time, defined as the time that an application may continue to operate without receiving an anticipated message [27]. The distribution of AoI with packet preemption and memoryless servers was investigated in [28]. In [29], the Chernoff bound was used to derive an upper bound of the quantile function of the AoI for two queues in tandem with deterministic arrivals. The peak-age violation probability, defined as the probability of exceeding a pre-defined PAoI threshold, was derived for a single-hop link with fading and retransmissions, in the form of variable-length-stop-feedback [3].

So far, only a few studies considered the impact of physical layer and medium access control on the AoI. Among these, recent works compute the average AoI in Carrier Sense Multiple Access (CSMA) [30], ALOHA [31], and slotted ALOHA [32] networks, considering the impact of the different medium access policies on the age. Of special interest for our study, the AoI with a TDMA-like scheme with perfect feedback and immediate retransmissions was compared to that of ALOHA [33]. It was observed that TDMA with retransmissions greatly reduced the AoI when compared to ALOHA. However, the former scheme assumes that the transmissions from all users after a transmission failure are delayed to allow for a retransmission to occur in the next time slot, which is inefficient, as a separate channel is needed for feedback.

There are only a few studies on heterogeneity in AoI systems. We mention the work presented in [34], which considered different service classes, and modeled the system as an M/G/1/1 queue with hyperexponential service time. However, only the service rate was different among classes. Then, the classes could adapt the arrival rate to minimize the AoI.

## 3. System Model

In the following, we denote random variables with capital letters (e.g., *X*) and their values with the corresponding lowercase letters (e.g., *x*). Sets are denoted in calligraphic font (e.g., U), and the corresponding standard capital letters denote their cardinality (e.g., *U*). Vectors are denoted with bold lowercase letters (e.g., x), and matrices with bold capital letters (e.g., X). probability mass functions (pmfs) are denoted with a lowercase *p* and Cumulative Distribution Functions (CDFs) with a capital *P*. Table 1 provides a quick reference for the most important notation used in the rest of the paper.

We define the outcome of the user’s activity in a slot as an event, which happens with probability *p* and is mutually exclusive with other outcomes. The outcome vector k then corresponds to the composite event in which the *i*-th outcome is observed $k_{i}$ times, and the probability vector p contains the probability of each outcome (which does not necessarily sum to 1 as we consider that none of the outcomes might occur). We can then define the multinomial function Mult(k;n,p), which corresponds to the probability of outcome vector k being observed over *n* slots.

(1) $$\mathrm{Mult}\left(k;n,p\right)=\frac{n!\prod_{i=1}^{\left|p\right|}p_{i}^{k_{i}}{\left(1-\sum_{i=1}^{\left|p\right|}p_{i}\right)}^{n-\sum_{i=1}^{\left|p\right|}k_{i}}}{\left(n-\sum_{i=1}^{\left|p\right|}k_{i}\right)!\prod_{i=1}^{\left|p\right|}k_{i}!},$$

where |p| is the length of vector p. The binomial function Bin(k;n,p) is the special case in which |k|=|p|=1.

We also define the modulo function, which behaves as expected from integer arithmetic.

(2) $$\mathrm{mod}\left(m,n\right)=m-\left\lfloor \frac{m}{n}\right\rfloor$$

for $m,n\in Z^{+}$. $Z^{+}$ is the set of non-negative integers.

### 3.1. Access Model

We consider an uplink scenario with a set of users U transmitting data to a Base Station (BS) over a single time-slotted multiple access channel. This single channel may consist of a single or of multiple subcarriers in an OFDMA system, whose number remains constant throughout the operation of the system. Users can transmit up to one packet per time slot, denoted by the index t∈Z, by occupying the available bandwidth and the entire duration of the slot. This can achieved by selecting a proper modulation and coding scheme based on the size of the payload to transmit. The study of multi-channel settings is considerably more complex, and left to future work, as having multiple concurrent resources in frequency domain changes the timing considerations significantly.

There is as set of users U in the system, composed of a single broadband user and multiple intermittent users. Specifically, user $u_{B}$ is the broadband user following the eMBB model: it is a full-buffer user that always has data to transmit and maintains an infinite transmission queue. To counteract potential packet losses due to the noise, the broadband user implements a packet-level coding scheme, where blocks of *K* source packets are encoded to generate a frame of *N* coded packets of length *ℓ* bits each. The basic operation of the broadband user is shown in Figure 1. The coded packets are linearly independent, which can be achieved, for example, with Maximum Distance Separable (MDS) codes or with Random Linear Network Coding (RLNC) with Galois-field size equal to *∞*. In effect, decoding any subset of *K* coded packets is sufficient for recovering the original block.

The intermittent users belong to the subset $U_{I}=U\backslash \left\{u_{B}\right\}$, where $U_{I}=U-1$. They generate packets in each slot with a probability α (i.e., they experience Bernoulli arrivals with parameter α) and maintain a queue of up to *Q* generated packets. If a new packet is generated when the instantaneous length of the queue is *Q*, these users discard the oldest buffered packet and add the newly generated one at the end of the queue. The choice of discarding the oldest packet in the queue follows a simple rationale: discarding any of the packets has the same effect on the overall reliability, choosing the oldest minimizes the latency for the ones that are delivered, as they will spend less time waiting for a slot in which they can be transmitted. In most practical cases, the queue will be set up so as to minimize the probability of discarding packets, but the case with short queues is relevant for low-power IoT devices with limited memory and computational resources. Packets are transmitted from the queue using First-In First-Out (FIFO) discipline, and the transmissions take place in the allocated slots.

We consider a static allocation scheme, in which users are synchronized at the slot level. The set of users that are allocated slot *t* is denoted by $A_{t}$, where $A_{t}\subseteq U$ s.t. $A_{t}\neq \emptyset$. We define the following three types of slot allocations.

1. *Broadband:* The slot is reserved for the broadband user. Hence, $A_{t}=\left\{u_{B}\right\}$.
2. *Intermittent:* The intermittent users are allocated the slot and may use it if there are packets in their queues. Hence, $A_{t}\subseteq U_{I}$.
3. *Mixed:* Both types of users are allowed to access the slot. Hence, $A_{t}\subseteq U$ s.t. $u_{B}\in A_{t}$ and $|A_{t}|>1$.

Next, we define the OMA and NOMA slicing based on the resource allocation as follows.

1. *OMA*: Slots can be either allocated to the broadband user or intermittent users; we define $T_{\mathrm{int}}$ to be the period between intermittent slots.
2. *NOMA*: Only mixed slots are allocated.

Finally, based on the allocation in the intermittent and mixed slots, we define the following three subdivisions of OMA and NOMA slicing. We take a slot *t* in which the intermittent users can transmit, i.e., any slot in NOMA or one of the intermittent slots in OMA.

1. *TDMA:* The slot is allocated to a single intermittent user, such that $|A_{t}\backslash \left\{u_{B}\right\}|=1$.
2. *Grouping:* The slot is allocated to $G\in \{2,\ldots ,U_{I}-1\}$ intermittent users s.t. $|A_{t}\backslash \left\{u_{B}\right\}|=G$ for all *t*. We consider the case where $\left(U_{I}\;\mathrm{mod}\;G\right)=0$, i.e., we can divide the intermittent users into groups of equal size.
3. *ALOHA:* All the intermittent users are allowed to transmit in the slot. Hence, $|A_{t}\backslash \left\{u_{B}\right\}|=U_{I}$ for all slots, excluding the broadband slots in OMA.

**Figure 2:**
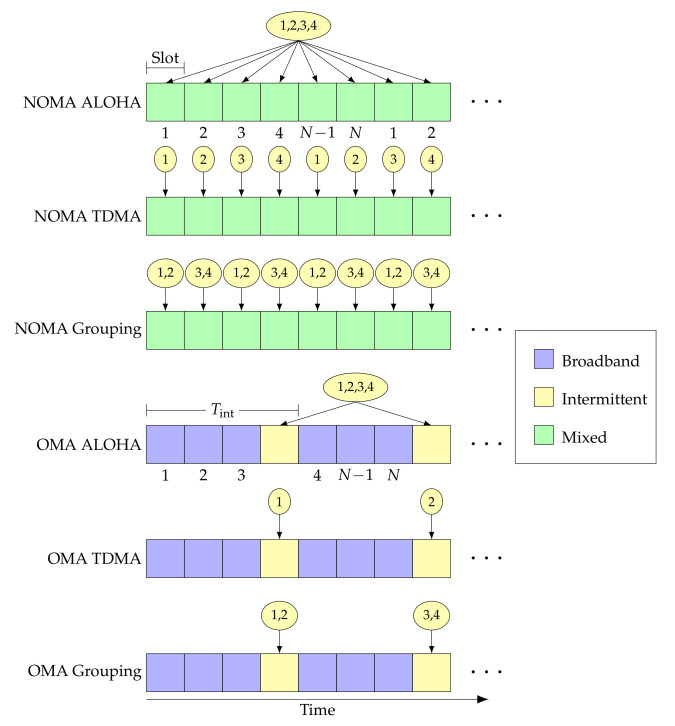
Frame structure for the considered access schemes with K=4, N=6, and U=4.

The frame structures for the six access schemes resulting from the combining of the slicing and allocation methods described above are illustrated in Figure 2: the circles represent the intermittent users that have access in any given slot, and the color of the square represents the type of access in that slot. We also not that the grouping scheme can be easily extended to cover the two extreme cases in which (i) there is only one group comprising all intermittent users (which is equivalent to ALOHA) and (ii) there is one user per group (which is equivalent to TDMA). Thus, it represents a general scheme which we can apply within OMA or NOMA.

### 3.2. Channel Model

We consider a quasi-static block fading channel, where the received signal by the BS at any slot *t* is given as

(3) $$y_{t}=\sum_{u\in U}h_{u,t}\;a_{u,t}\;x_{u,t}+z_{t},$$

where $h_{u,t}$ is the random fading coefficient for user *u* at slot *t* and $z_{t}$ is an Additive White Gaussian Noise (AWGN) noise with variance $\sigma^{2}$. The random variable $a_{u,t}\in \left\{0,1\right\}$ models user’s activity, being equal to 1 if the user is active in that slot and 0 otherwise. A user is active only if it is allowed to transmit; i.e., if $u\in A_{t}$, and if its packet queue $q_{u,t}$ is not empty:

(4) $$a_{u,t}=I\left(u\in A_{t}\right)I\left(q_{u,t}>0\right),$$

where I(×) is the indicator function, equal to 1 if the condition is true and 0 otherwise. Let $P_{u}$ be the fixed transmission power of user *u*, which can be different for each user. The Signal to Noise Ratio (SNR) of user *u* at time slot *t* is given by:

(5) $$\mathrm{SNR}\left(u,t\right)=\frac{|h_{u,t}{|}^{2}P_{u}\;a_{u,t}}{|z_{t}{|}^{2}},$$

whereas the Signal to Interference plus Noise Ratio (SINR) of user *u* at time slot *t* is given by

(6) $$\mathrm{SIN}R\left(u,t\right)=\frac{|h_{u,t}{|}^{2}P_{u}\;a_{u,t}}{|z_{t}{|}^{2}+\sum_{v\in U\backslash \{u\}}{\left|h_{v,t}\right|}^{2}P_{v}\;a_{v,t}},$$

where U\u is the set of users except user *u*. We can also simply divide the SINR by the noise power $|z_{t}{|}^{2}$, giving

(7) $$\mathrm{SIN}R\left(u,t\right)=\frac{\mathrm{SNR}(u,t)}{1+\sum_{v\in U\backslash \{u\}}\mathrm{SNR}\left(v,t\right)}.$$

Hence, the SINR is equal to the SNR in the absence of interference. Next, we define γ as the threshold in the SNR to decode a packet. That is, γ defines the erasure probability of a binary erasure channel (BEC) as

(8) $$\varepsilon_{u}=\mathrm{Pr}\left[\mathrm{SNR}(u,t)<\gamma \right]\;\forall t,u:a_{u,t}=1.$$

Further, we consider a simple collision model, so that packets cannot be decoded in the presence of interference (i.e., collisions). Hence, a packet from user *u* can be decoded, with probability $\left(1-\varepsilon_{u}\right)$ if and only if SNR(u,t)=SINR(u,t). This model neither allows for capture, nor for potential subsequent application SIC within slots containing more than one transmission (i.e., intra-collision SIC), representing the worst-case scenario for schemes that rely on MPR, such as power-domain NOMA. Instead, SIC can be only performed after decoding the broadband user, regeneration of all its *N* coded packets, and removing them from the slots that also contain transmissions from the intermittent users (i.e., extra-collision SIC). In slots without a collision, we assume a constant erasure probability for each user, denoted as $\varepsilon_{B}$ for the broadband user and $\varepsilon_{I}$ for the intermittent user. Our assumption is that the erasure probability after the interference is canceled is the same as for a free channel, which is a simplification. However, the use of parity checks on all the packets in a frame means that the probability of erroneous packet decodings is very low, and modeling the precise performance of SIC schemes is beyond the scope of this paper, and this is a common assumption in the coded slotted ALOHA literature, which assumes a similar setting [35]. The model provides a general view on the lower bound on performance of the OMA and NOMA schemes that is independent of the underlying channel model.

### 3.3. Key Performance Indicators

The KPIs of interest are described in the following.

We first define the AoI ξ, which in our case is the number of slots that have passed since the generation of the last correctly received packet. If packet *i* is generated in slot $g_{i}$ and decoded by the receiver in slot $d_{i}$, while packet i+1 is generated in slot $g_{i+1}>g_{i}$ and decoded by the receiver in slot $d_{i+1}>d_{i}$, we have:

(9) $$\xi \left(n\right)=n-g_{i},\;\forall n\in \left\{d_{i},\ldots ,d_{i+1}-1\right\}.$$

The PAoI Δ is then simply defined as the AoI, measured at the instant of arrival of a new packet:

(10) $$\Delta_{i}=\xi \left(d_{i}\right).$$

**Figure 3:**
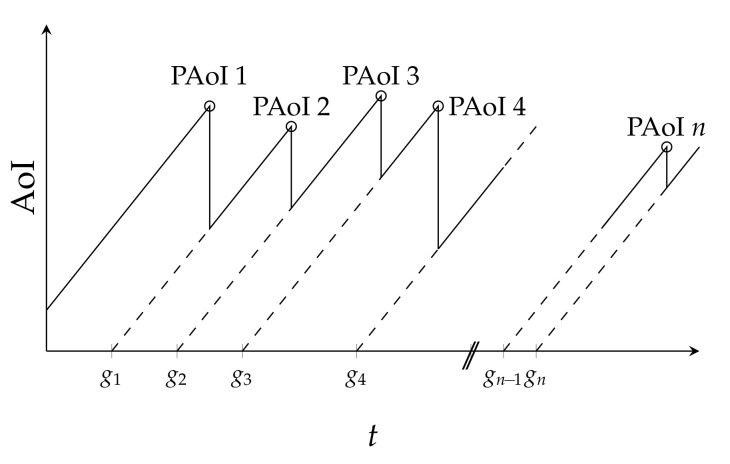
Evolution of the AoI and PAoI.

The PAoI is the maximum value of the AoI across a cycle, as depicted in Figure 3.

The relevant KPI for PAoI-oriented systems is the 90th percentile of the PAoI, denoted by $\Delta_{90}$.

(11) $$\Delta_{90}=\min_{n\in Z^{+}}\left\{n:\mathrm{Pr}\left[\Delta \leq n\right]\geq 0.9\right\},$$

Latency and age are expressed in slots. $\Delta_{90}$ allows us to assess the tail distribution of the PAoI in a general scenario, and can be used to compare performance with different values of the slot arrival rate α. In contrast, a widely employed metric called *PAoI violation probability* [3] requires the definition of a specific threshold, either expressed as an absolute time or as a maximum number of slots. Furthermore, it cannot be used to compare the performance under different arrival rates α since the AoI is greatly determined by the latter.

For latency-oriented systems, we introduce a similar KPI, which is the 90th percentile of the latency-reliability for intermittent users. The distribution of latency-reliability is computed by multiplying the distribution of the latency of successfully received packets by their success probability $p_{s,I}$:

(12) $$T_{90}=\min_{n\in Z^{+}}\left\{n:\mathrm{Pr}\left[T\leq n\right]p_{s,I}\geq 0.9\right\},$$

on all packets, not just the successfully delivered ones,

We can now define the Pareto frontier, which is commonly used in multi-objective optimization:

**Definition** **1.** *Let $f:{\left(Z^{+}\right)}^{2}\to R\times Z^{+}$ and C be the set of feasible configurations. Next, let*

$$Y=\{\left(S_{B},\tau \right):\left(S_{B},\tau \right)=f\left(c\in C\right),\},$$

*where $S_{B}$ is the throughput of the broadband user and τ is the timeliness of the intermittent user, i.e., $\Delta_{90}$ or $T_{90}$. The Pareto frontier is the set*

(13) $$P\left(Y\right)=\{\left(S_{B},\tau \right)\in Y:\left\{\left(S_{B}^{\prime },\tau^{\prime }\right)\in Y:S_{B}>S_{B}^{\prime },\tau <\tau^{\prime }\right\}=\emptyset \}.$$

## 4. Orthogonal Multiple Access

We first consider algorithms based on OMA, assuming that $U_{I}>1$, and, for the sake of simplicity, that all intermittent users have the same slot arrival rate α. In an OMA system, the broadband user transmits in frames of *N* broadband slots, each of which contains an encoded data packet. It is sufficient to decode *K* of the *N* packets to recover the whole frame. Reserved slots for the intermittent users are interleaved with the ones for the broadband user: there is one intermittent slot every $T_{\mathrm{int}}$, where in general $T_{\mathrm{int}}\neq N$, and in which one or more intermittent users try to access the channel.

### 4.1. PAoI-Oriented System

In PAoI-oriented OMA system the transmission queue size is Q=1 and preemptive scheduling is used, i.e., a newly arrived packets replaces the one stored in the buffer. In this case, the KPIs are given by $\Delta_{90}$ (the 90th percentile of the PAoI) for the intermittent users, and the throughput $S_{B}$ for the broadband user. We consider the grouping model, in which the $U_{I}$ intermittent users are divided into *G* groups. As we mentioned earlier, the scheme applies TDMA between groups. Users in the same group contend for the channel in the same slots. The ALOHA and TDMA systems are extreme cases of the grouping scheme, with G=1 and $G=U_{I}$, respectively. The OMA grouping scheme is represented in Figure 2, along with the two extreme cases.

Denote the probability of successfully decoding the broadband users frame (i.e., the *N* packets contained in it) as $p_{s,B}$. The throughput $S_{B}$ is

(14) $$S_{B}=p_{s,B}\frac{(T_{\mathrm{int}}-1)K}{T_{\mathrm{int}}N}.$$

As the broadband user can only use $T_{\mathrm{int}}-1$ slots out of every $T_{\mathrm{int}}$, setting up more frequent transmission opportunities for the intermittent users reduces the broadband user’s throughput. Probability $p_{s,B}$ is easy to compute in this case, as orthogonal access prevents collisions with the intermittent users.

(15) $$p_{s,B}=\sum_{r=K}^{N}\mathrm{Bin}\left(r:N,1-\varepsilon_{B}\right).$$

In order to compute the success probability for intermittent users, we first consider the probability ρ that an intermittent user accesses the channel in the next intermittent slot that is allocated to it, i.e., the probability that at least one packet is generated in a $GT_{\mathrm{int}}$ interval.

(16) $$\rho =1-{\left(1-\alpha \right)}^{GT_{\mathrm{int}}}.$$

The probability $p_{s,I}$ that a packet from an intermittent user is decoded successfully is then given by two components. First, all other intermittent users in the same group must not have any packets to send in that slot, and second, there must not be a channel erasure.

(17) $$p_{s,I}=\left(1-\varepsilon_{I}\right){\left(1-\rho \right)}^{\frac{U_{I}}{G}-1}.$$

The PAoI is then Δ=W+Z, given by the sum of two components: the first, *W*, is the waiting time between the generation of a packet and its successful transmission. The second, *Z*, is the inter-transmission time between the slot when the packet is transmitted and the slot in which the next successful packet from the same user is decoded.

The pmf of the waiting delay *W* of a successful transmission in PAoI-oriented OMA is then given by:

(18) $$p_{W}\left(w\right)=\frac{\alpha {\left(1-\alpha \right)}^{w}}{1-{\left(1-\alpha \right)}^{GT_{\mathrm{int}}}},\;w\in \left\{0,\ldots ,GT_{\mathrm{int}}-1\right\}.$$

Since transmission opportunities for the intermittent users in a given group are scheduled in one slot every $GT_{\mathrm{int}}$, *Z* is $GT_{\mathrm{int}}$ times the number of reserved slots between consecutive transmissions. This is a geometric random variable, whose parameter is $\rho p_{s,I}$. The pmf of *Z* is then given by

(19) $$p_{Z}\left(z\right)={\left(1-\rho p_{s,I}\right)}^{\frac{z}{GT_{\mathrm{int}}}-1}\rho p_{s,I},\;\forall z\in Z^{+}\backslash \left\{0\right\}:\mathrm{mod}\left(z,GT_{\mathrm{int}}\right)=0.$$

The pmf of the PAoI Δ is now easy to find by convolving the distributions of *W* and *Z*. Since *W*’s support is $\{0,\ldots ,GT_{\mathrm{int}}-1$, and *Z*’s support is $GT_{\mathrm{int}}\times Z^{+}$, the convolution is reduced to a simple multiplication:

(20) $$p_{\Delta }\left(\tau \right)=p_{W}\left(\mathrm{mod}(\tau ,GT_{\mathrm{int}})\right)p_{Z}\left(\tau -\mathrm{mod}(\tau ,GT_{\mathrm{int}})\right),\forall \tau \in Z^{+}.$$

We can now easily derive the KPI $\Delta_{90}$ by applying (11).

### 4.2. Latency-Oriented System

We now examine the relevant KPIs for the latency-oriented case. In this case, intermittent users maintain a queue of up to Q≥1 packets, discarding the oldest one when a new packet arrives and the queue is already full. As for the PAoI case, we consider the grouping system, in which the $U_{I}$ intermittent users are placed in *G* groups. The throughput of the broadband user is the same as in the PAoI-oriented system, given by (14). We now focus on intermittent user *u*: the state of its queue is represented by a Markov chain, whose discrete time instants represent the time just after each slot allocated to it. In the following, we will refer to any slot allocated to the considered user *u* as an allocated slot. The elements of the state transition probability matrix $P^{\left(Q\right)}$ are given by

(21) $$P_{ij}^{\left(Q\right)}=\left\{\begin{array}{cc} 0, & \mathrm{if}\;j<i-1;\\ \mathrm{Bin}(j-i+1;GT_{\mathrm{int}},\alpha ), & \mathrm{if}\;i-1\leq j<Q-1;\\ \sum_{k=Q-i+1}^{GT_{\mathrm{int}}}\mathrm{Bin}\left(k;GT_{\mathrm{int}},\alpha \right), & \mathrm{if}\;j=Q-1. \end{array}\right.$$

Using basic Markov theory, the steady-state distribution $\pi^{\left(0\right)}$ is derived as the left-eigenvector of $P^{\left(Q\right)}$ with eigenvalue 1, normalized to sum to 1.

(22) $$\left\{\begin{array}{cc} \; & \pi^{\left(0\right)}\left(I-P^{\left(Q\right)}\right)=0;\\ \; & \sum_{q=0}^{Q}\pi_{q}^{\left(0\right)}=1. \end{array}\right.$$

We can now consider the slots between two allocated slots by deriving the steady-state distribution *n* slots after the last allocated slot, which we denote as $\pi^{\left(n\right)}$.

(23) $$\pi_{i}^{\left(n\right)}=\left\{\begin{array}{cc} \sum_{j=0}^{i}\pi_{i}^{\left(0\right)}\mathrm{Bin}\left(i-j;n\alpha \right), & \mathrm{if}\;i<Q;\\ \sum_{j=0}^{Q}\sum_{k=Q-j}^{n}\pi_{j}^{\left(0\right)}\mathrm{Bin}\left(k;n,\alpha \right), & \mathrm{if}\;i=Q. \end{array}\right.$$

At each allocated slot, the oldest packet in the queue is transmitted. If a new packet is generated when the queue is already full, the oldest packet is dropped from the buffer. Consider a specific packet generated in the *n*-th slot after an allocated one: if it finds *q* packets in the queue when it is generated, it will be transmitted at the q+1-th allocated slot after it is generated, unless some packets ahead of it are dropped due to new arrivals. We can then define a generation vector of length *ℓ*, whose *i*-th element contains the number of packets generated in the slots between the i−1-th and *i*-th allocated slots after the generation of the considered packet. The first element of the vector contains the number of packets generated between the considered packet’s generation and the first allocated slot after it. We then define the set $G_{\ell }^{\left(n\right)}$, which contains all the generation vectors of length *ℓ* for a packet generated in the *n*-th slot after the last one allocated:

(24) $$G_{\ell }^{\left(n\right)}=\left\{0,\ldots ,GT_{\mathrm{int}}-n\right\}\times {\left\{0,\ldots ,GT_{\mathrm{int}}\right\}}^{\ell -1}.$$

The probability of each generation vector in the set is then given by:

(25) $$p_{\mathrm{gen}}\left(g;\ell ,n\right)=\mathrm{Bin}\left(g_{1};GT_{\mathrm{int}}-n,\alpha \right)\prod_{i=2}^{\ell }\mathrm{Bin}\left(g_{i};GT_{\mathrm{int}},\alpha \right).$$

The considered packet is then transmitted by the *ℓ*-th allocated slot after its generation if q+1−ℓ packets ahead of it are either dropped or transmitted at that point. For a given generation vector $g\in G_{\ell }^{\left(n\right)}$, we then formulate condition $\psi_{\ell }^{\left(g,q\right)}$:

(26) $$\psi_{\ell }^{\left(g,q\right)}=\delta \left(\sum_{i=1}^{\ell }{\left[q+1-Q+\sum_{j=1}^{i}g_{j}\right]}^{+}+\ell -\left(q+1\right)\right),$$

where δ(x) is the delta function, which is equal to 1 if x=0 and 0 otherwise, and ${\left[x\right]}^{+}=\max\left(x,0\right)$. The condition naturally excludes the cases in which the considered packet is dropped, i.e., when a new packet arrives and finds a full queue, with the considered packet being first in line. We can then define the set $H_{\ell }^{\left(n,q\right)}$, which contains the elements $g\in G_{\ell }^{\left(n\right)}$ for which the considered packet is transmitted at the *ℓ*-th opportunity:

(27) $$H_{\ell }^{\left(n,q\right)}=\left\{g\in G_{\ell }^{\left(n\right)}:\psi_{\ell }^{\left(g,q\right)}-\sum_{k=1}^{\ell -1}\psi_{k}^{\left(g,q\right)}=1\right\}.$$

The maximum value of *ℓ* is q+1, as by that point the packet has either been transmitted or dropped. Consequently, the success probability $p_{s,I}\left(n,q\right)$ for an intermittent user arriving *n* slots after an allocated one and finding a queue of *q* packets ahead of it is given by

(28) $$\begin{array}{c} p_{s,I}\left(n,q\right)=\sum_{\ell =1}^{q+1}\sum_{g\in H_{\ell }^{\left(n,q\right)}}p_{\mathrm{gen}}\left(g;\ell ,n\right)\left(1-\varepsilon_{I}\right)p_{\mathrm{free}}. \end{array}$$

The packet can only be received correctly if the channel is free and there are no channel errors, as we assume totally destructive interference. The probability of having a free channel is equal to the probability that none of the other intermittent users in the same group have any packets in their queues:

(29) $$p_{\mathrm{free}}={\left(\pi_{0}^{\left(n-1\right)}\right)}^{\frac{U_{I}}{G}-1}$$

We can then compute the conditioned latency distribution:

(30) $$p_{T}\left(\ell GT_{\mathrm{int}}-n;q,n\right)=\frac{\sum_{g\in H_{\ell }^{\left(n,q\right)}}p_{\mathrm{gen}}\left(g;\ell ,n\right)}{p_{s,I}\left(n,q\right)}.$$

Knowing that packet generation probability is the same for every slot, we can now use (23) to derive the overall success probability.

(31) $$p_{s,I}=\sum_{n=1}^{T_{\mathrm{int}}}\sum_{q=0}^{Q}\frac{\pi_{q}^{\left(n-1\right)}p_{s,I}\left(n,q\right)}{GT_{\mathrm{int}}}.$$

In the same way, we derive the latency pmf:

(32) $$p_{T}\left(t\right)=\sum_{n=1}^{GT_{\mathrm{int}}}\sum_{q=0}^{Q}\frac{\pi_{q}^{\left(n-1\right)}p_{T}\left(t;\min\left(q,Q-1\right),n\right)p_{s,I}\left(n,q\right)}{GT_{\mathrm{int}}p_{s,I}}.$$

The 90th percentile of the packet delivery latency $T_{90}$ can be derived by applying the definition in (12).

## 5. Non-Orthogonal Multiple Access

We now examine the performance of NOMA schemes, in which the intermittent users’ packets can collide with the broadband user’s packets, and among themselves. If the broadband user frame (i.e., *N* packets contained in it) has been recovered, the receiver performs SIC to remove the broadband user’s packets from the slots. In the next step, the receiver attempts decoding intermittent users’ packets which may be contained in the slots affected by SIC. According to the channel model, the decoding succeed only if there was a single intermittent user transmission (i.e., packet) in a slot, and it was not affected by a channel erasure. As for the OMA case, we consider the grouping case. In this case, each intermittent user can transmit once every *G* slots, along with the other users in the same group.

### 5.1. PAoI-Oriented System

As in the OMA case, we first consider a PAoI-oriented system, in which Q=1 and preemptive scheduling are used for all intermittent users. Since all intermittent users have the same arrival rate α, we can easily compute the success probability of the broadband user:

(33) $$p_{s,B}=\sum_{r=K}^{N}\mathrm{Bin}\left(r;N,\left(1-\varepsilon_{B}\right){\left(1-\alpha \right)}^{U_{I}}\right).$$

The throughput for the broadband user is then:

(34) $$S_{B}=\frac{Kp_{s,B}}{N}.$$

We now turn to computing the value of $\Delta_{90}$. We consider a specific intermittent user *u*, whose probability of generating at least one packet before the next allocated slot is

(35) $$\rho =1-{\left(1-\alpha \right)}^{G}.$$

If a packet from an intermittent user is transmitted, the probability of success (without considering the interference from the broadband user) is

(36) $$p_{s,I}=\left(1-\varepsilon_{I}\right){\left(1-\rho \right)}^{\frac{U_{I}}{G}-1}.$$

As we did in the OMA case, we can divide the PAoI in three parts:

(37) Δ=W+Y+Z,

where, as above, *W* is the waiting time from the packet generation to its transmission and *Z* is the inter-transmission time. *Y* is the decoding latency, i.e., the number of slots from the transmission until its successful decoding, which is 0 for OMA (in that case, packets are either decoded immediately or lost due to erasure or collision), but can be non-zero for NOMA if the packet is recovered later with SIC. The distribution of *W* is simple to derive:

(38) $$p_{W}\left(w\right)=\frac{\alpha {\left(1-\alpha \right)}^{w}}{\rho },\;w\in \left\{0,\ldots ,G-1\right\}.$$

We can now compute the pmfs of *Y* and *Z*, but to do so we first compute some auxiliary functions. We define the offset *o* as the index of the slot that represents the first allocated slot for the considered user in the frame. Denote by $T_{o}\left(d\right)$ the set of transmission opportunities for the user from the beginning of the frame to slot *d*, whose first element is *o*:

(39) $$T_{o}\left(d\right)=\left\{i\in \{o,\ldots ,d\}:\mathrm{mod}(i-o,G)=0\right\}.$$

The probability that the user will transmit *m* packets by slot *d* for a given offset *o* is

(40) $$p_{\mathrm{tx}}\left(m;d,o\right)=\mathrm{Bin}(m;|T_{o}\left(d\right)\left|,\rho \right).$$

We now derive the probability that the first packet from the intermittent user to be decoded in a frame is correctly received in slot *d*. This only happens if three conditions are met:

1. The interference from the broadband user can be successfully removed by SIC; i.e., *K* packets from it have been received and decoded in the current frame.
2. There is no interference from other intermittent users.
3. There are no channel errors.

The second and third conditions are easy to compute, and are summarized by (36). To consider the third one, we consider the two cases in which the packet is transmitted and decoded in the same slot (denoted as A) and the one in which it is decoded later (denoted as B). In the former case, at least *K* packets from the broadband user have already arrived before *d*, and SIC is performed immediately; in the latter case, the intermittent user packet is retroactively decoded when the *K*-th broadband user packet is decoded.

We start with the first one:

(41) $$\begin{array}{c} p_{F}\left(d;o|A\right)=\rho p_{s,I}\sum_{m=0}^{|T_{o}\left(d\right)|}p_{\mathrm{tx}}\left(m;d,o\right)\mathrm{Bin}\left(0;m,p_{s,I}\right)\sum_{r_{o}=0}^{d-|T_{o}\left(d\right)|}\mathrm{Bin}\left(r_{o};\left|T_{o}\left(d\right)\right|-m,\left(1-\varepsilon_{B}\right){\left(1-\rho \right)}^{\frac{U_{I}}{G}}\right)\\ \;\;\;\;\;\;\;\;\;\;\;\;\;\;\;\;\times \sum_{r_{t}=K-r_{o}}^{|T_{o}\left(d\right)|-m}\mathrm{Bin}\left(r_{t};\left|T_{o}\left(d\right)\right|-m,\left(1-\varepsilon_{B}\right){\left(1-\rho \right)}^{\frac{U_{I}}{G}-1}\right). \end{array}$$

In case A, the decoding delay is always 0, i.e., Y=0. In case B, the probability of a packet from the intermittent user being decoded in slot *d* is equivalent to the probability of at least one packet from the user being transmitted in the frame, and the *K*-th packet from the broadband user is decoded in slot *d*.

(42) $$\begin{array}{c} p_{F}\left(d;o|B\right)=\rho p_{s}^{\left(I\right)}\sum_{m=1}^{|T_{o}\left(d\right)|}p_{\mathrm{tx}}\left(m-1;d,o+G\right)\sum_{r=0}^{|T_{o}\left(d\right)|-m}\mathrm{Bin}\left(r;|T_{o}\left(d\right)|-m,\left(1-\varepsilon_{B}\right){\left(1-\alpha \right)}^{\frac{U_{I}}{G}-1}\right)\\ \;\;\;\;\;\;\times \left(1-\varepsilon_{B}\right){\left(1-\alpha \right)}^{U_{I}}\mathrm{Bin}\left(K-1-r;d-|T_{o}\left(d\right)|-1,\left(1-\varepsilon_{B}\right){\left(1-\alpha \right)}^{U_{I}}\right). \end{array}$$

The pmf of the decoding delay *Y* in case B is more complicated.

(43) $$\begin{array}{c} p_{Y}\left(y;d,o|B\right)=\sum_{c=1}^{|T_{o}\left(d\right)|}{\left(1-\alpha \right)}^{U_{I}}\left(1-\varepsilon_{B}\right)\sum_{e=0}^{|T_{o}\left(d\right)|-c}\mathrm{Mult}(\left(c,e\right);\left|T_{o}\left(d\right)\right|,\left(\rho p_{s,I},\rho \left(1-p_{s,I}\right)\right)\\ \times \frac{|T_{o}\left(d\right)|!(|T_{o}\left(d-y\right)\left|-c-1\right)!}{c|T_{o}\left(d-y\right)|!}\rho p_{s,I}\sum_{r=0}^{|T_{o}\left(d\right)|-m}\mathrm{Bin}(r;|T_{o}\left(d\right)\left|-m,\left(1-\varepsilon_{B}\right){\left(1-\alpha \right)}^{\frac{U_{I}}{G}-1}\right)\\ \;\;\;\;\;\times \mathrm{Bin}(K-1-r;d-|T_{o}\left(d\right)\left|-1,\left(1-\varepsilon_{B}\right){\left(1-\alpha \right)}^{U_{I}}\right),\;d-y\in T_{o}\left(d\right). \end{array}$$

If *d* is an allocated slot, we have to consider both cases, but if it is not, the only possible case is the first one.

(44) $$p_{F}\left(d;o\right)=\left\{\begin{array}{cc} p_{F}\left(d;o|A\right)+p_{F}\left(d;o|B\right) & \mathrm{if}\;d\in T_{o}\left(d\right);\\ p_{F}\left(d;o|B\right) & \mathrm{if}\;d\notin T_{o}\left(d\right). \end{array}\right.$$

We can then compute the probability that no packets will be delivered in a frame with a given offset.

(45) $$p_{⌀}\left(o\right)=1-\sum_{d=K+1}^{N}p_{F}\left(d;o\right).$$

Naturally, the delay of decoding events that come after the first in the frame is always 0, as SIC can instantly decode the packet from the intermittent user. We can now compute the probability of having a decoding event in a given slot *d*, given that the first decoding event was in slot *f* and the offset is *o*.

(46) $$p_{R}\left(d;f,o\right)=\left\{\begin{array}{cc} 1 & \mathrm{if}\;d=f;\\ \rho p_{s,I} & \mathrm{if}\;d\in T_{o}\left(N\right)\wedge d>f;\\ 0 & \mathrm{otherwise}. \end{array}\right.$$

We can then uncondition on *f* and get $p_{R}\left(d\right)$:

(47) $$p_{R}\left(d\right)=\sum_{f=K+1}^{d}p_{R}\left(d;f,o\right)p_{F}\left(f;o\right).$$

With this, we compute the probability that a decoding event in a given slot is the first in the frame:

(48) $$p_{E}\left(d;o\right)=\frac{p_{F}\left(d;o\right)}{p_{R}\left(d;o\right)\left(1-p_{N}\left(d;o\right)\right)}.$$

We can then compute the pmf of the latency *T* for a decoding in slot *d*.

(49) $$p_{Y}\left(y;d,o\right)=\left\{\begin{array}{cc} p_{E}\left(d;o\right)+\left(1-p_{E}\left(d;o\right)\right)\frac{p_{F}\left(d;o|A\right)}{p_{F}\left(d;o\right)} & \mathrm{if}\;y=0,d\in T_{o}\left(d\right);\\ p_{E}\left(d;o\right) & \mathrm{if}\;y=0,d\notin T_{o}\left(d\right);\\ \left(1-p_{E}\left(d;o\right)\right)p_{Y}\left(y;d,o|B\right) & \mathrm{if}\;y>0. \end{array}\right.$$

The final component of the PAoI is the inter-arrival time, *Z*. There are two separate cases for this: either the two consecutive decoding events are in the same frame, or the next one is in a future frame. We first find the probability that a given decoding event is the last in the frame:

(50) $$p_{L}\left(d;o\right)={\left(1-\rho p_{s,I}\right)}^{|T_{o}\left(N\right)|-|T_{o}\left(d\right)|}.$$

If the next packet from the intermittent user is in the same frame, we have:

(51) $$p_{Z}\left(z;d,o,\bar{L}\right)=\frac{\rho p_{s,I}{\left(1-\rho p_{s,I}\right)}^{\frac{z}{G}}}{1-p_{L}\left(d;o\right)},d+z\in \left(T_{o}\left(N\right)\backslash T_{o}\left(d\right)\right).$$

If the next packet is in a future frame, we need to compute the offset for the next frames. We denote the offset for the *i*-th frame after the current one, which has offset *o*, as $\omega_{i}\left(o\right)$.

(52) $$\omega_{i}\left(o\right)=\min\left(T_{o}\left(\left(i+1\right)N\right)\backslash T_{o}\left(iN\right)\right)-iN.$$

If the number of groups *G* is larger than the number of slots in a frame *N*, there might be no transmission opportunities in a frame; in that case, $T_{\omega_{i}\left(o\right)}\left(N\right)=\emptyset$, and the intermittent user will never transmit in that frame. For a given inter-transmission time *z*, we can then define the number of frames without successfully received intermittent packets as M(z;d,o):

(53) $$M\left(z;d,o\right)=\left\lfloor \frac{z+d-1}{N}-1\right\rfloor .$$

We can then give the pmf of the inter-transmission time if the next packet is not in the same frame:

(54) $$p_{Z}\left(z;d,o,L\right)=p_{F}\left(z-NM\left(z;d,o\right)+d;\omega_{M(z;d,o)+1}\left(o\right)\right)\prod_{i=0}^{M(z;d,o)}p_{⌀}\left(\omega_{i}\left(o\right)\right).$$

By unconditioning over *L* and *d*, we get the pmf of the inter-transmission time:

(55) $$p_{Z}\left(z;d,o\right)=\left\{\begin{array}{cc} \left(1-p_{L}\left(d;o\right)\right)p_{Z}\left(z;d,o,\bar{L}\right) & \mathrm{if}\;d+z\leq N;\\ p_{L}\left(d;o\right)p_{Z}\left(z;d,o,L\right) & \mathrm{if}\;d+z>N. \end{array}\right.$$

We can now join the results in (38), (49), and (55) to get the pmf of the PAoI for a given offset:

(56) $$p_{\Delta }\left(\tau ;o\right)=\sum_{d=k+1}^{N}p_{R}\left(d;o\right)\sum_{w=0}^{\min(G-1,\tau )}p_{W}\left(w\right)\sum_{y=0}^{\min(\tau -w,d)}p_{Y}\left(y;d,o\right)p_{Z}\left(\tau -w-y;d,o\right).$$

Finally, we uncondition on the offset *o* by considering all the possible offsets for a user. We assume that the initial offset is $o_{0}$, and denote the set of reachable offsets from $o_{0}$ as $O(o_{0})$.

(57) $$O\left(o_{0}\right)=\left\{o\in (\left\{1,\ldots ,G\right\}\wedge T_{o_{0}}\left(\infty \right))\right\}.$$

The probability of having a random decoded packet be in a frame with offset *o* is then given by

(58) $$p_{O}\left(o;o_{0}\right)=\frac{1-p_{⌀}\left(o\right)}{\sum_{o^{\prime }\in O\left(o_{0}\right)}1-p_{⌀}\left(o^{\prime }\right)}.$$

We can now uncondition the PAoI pmf:

(59) $$p_{\Delta }\left(\tau ,o_{0}\right)=\sum_{o\in O(o_{0})}p_{O}\left(o;o_{0}\right)p_{\Delta }\left(\tau ;o\right).$$

We remark that the grouping scheme is not necessarily fair to users, as users with a different initial index might have slightly different PAoI distributions.

### 5.2. Latency-Oriented System

We now derive the distributions of the KPIs in the NOMA latency-oriented case. As for OMA, intermittent users maintain a queue of up to *Q* packets, and we can define the transition matrix $P^{\left(Q\right)}$ of the Markov chain representing the queue state of an intermittent user right after two successive transmission opportunities:

(60) $$P_{ij}^{\left(Q\right)}=\left\{\begin{array}{cc} 0 & \mathrm{if}\;j<i-1;\\ \mathrm{Bin}(j-i+1;G,\alpha ) & \mathrm{if}\;i-1\leq j<Q-1;\\ \sum_{k=Q-i+1}^{G}\mathrm{Bin}\left(k;G,\alpha \right) & \mathrm{if}\;j=Q-1. \end{array}\right.$$

Using the same procedure as in (23), we can derive the steady-state distribution $\pi^{\left(0\right)}$, and then the value of $\pi^{\left(n\right)}$ in intermediate slots. We can then define the success probability and throughput for the broadband user

(61) $$\begin{array}{cc} p_{s,B} & =\sum_{r=K}^{N}\mathrm{Bin}\left(r;N,\left(1-\varepsilon_{B}\right){\left(\pi_{0}^{\left(G-1\right)}\right)}^{\frac{U_{I}}{G}}\right) \end{array}$$

(62) $$\begin{array}{cc} S_{B} & =\frac{Kp_{s,B}}{N}. \end{array}$$

We now analyze the latency for an intermittent user. As in the PAoI case, we consider an offset *o*, with a set of possible transmissions $T_{o}\left(N\right)$ given by (39). Latency is composed of two parts, the waiting time *W* and the decoding time *Y*. The waiting time is the time from the generation of the packet until it is transmitted, and the decoding time depends on when the frame from the broadband user is decoded. As it was done for the OMA case, we define the generation set $G_{\ell }^{\left(n\right)}$, which contains the possible numbers of arrivals in each transmission window after the generation of the considered one:

(63) $$G_{\ell }^{\left(n\right)}=\left\{0,\ldots ,G-n\right\}\times {\left\{0,\ldots ,G\right\}}^{\ell -1}.$$

The probability of each element in the set is given by:

(64) $$p_{\mathrm{gen}}\left(g;\ell ,n\right)=\mathrm{Bin}\left(g_{1};G-n,\alpha \right)\prod_{i=2}^{\ell }\mathrm{Bin}\left(g_{i};G,\alpha \right).$$

As we did for OMA, we define the set $H_{\ell }^{\left(n,q\right)}$, which contains the elements $g\in G_{\ell }^{\left(n\right)}$ for which the considered packet is transmitted at the *ℓ*-th opportunity, following the definitions we gave in (26) and (27). We compute the dropping probability for a packet generated in slot *n* with *q* packets ahead of it as such:

(65) $$p_{D}\left(n,q\right)=1-\sum_{\ell =1}^{q+1}\sum_{g\in H_{\ell }^{\left(n,q\right)}}p_{\mathrm{gen}}\left(g;\ell ,n\right).$$

We can now compute $p_{W}\left(w;n,q\right)$

(66) $$p_{W}\left(w;n,q\right)=\frac{\sum_{g\in H_{\left\lfloor \frac{w}{G}\right\rfloor }^{\left(n,q\right)}}p_{\mathrm{gen}}\left(g;\left\lfloor \frac{w}{G}\right\rfloor ,n\right)}{1-p_{D}\left(n,q\right)}\delta \left(w-G\left(\left\lfloor \frac{w}{G}\right\rfloor +1\right))+n\right).$$

In order to compute *Y*, we need to consider the fact that transmission opportunities before or after the one in which the packet is sent are used by the same user. We consider a packet generated in slot *i* in a frame with offset *o*, which finds *q* packets ahead of it and waits for *w* slots before being transmitted. If the transmission is in the same frame as the packet generation, there might be *C* transmission opportunities unused by the user before the packet generation, whereas if the transmission is in a subsequent frame, the user is active in all transmission opportunities in the frame before the one in which the packet is transmitted, because it still has packets in the queue. We know that the offset of the frame in which the packet is transmitted is $\omega_{\left\lfloor \frac{i+w}{N}\right\rfloor }\left(o\right)$, as given by (52). In the following, we will simply refer to this value as ω to simplify the notation. We then have that C=0 if i+w>N, and in the other case we need to consider the possible events that happened before the generation of the considered packet.

We now compute the pmf of *C*. There are $|T_{o}\left(i-1\right)|$ transmission opportunities before the generation of the packet. We define n(i;o) as the slots between the last available allocated slot and slot *i*:

(67) $$n\left(i;o\right)=i-\max(T_{o}\left(i-1\right)\cup \left\{o-G\right\}).$$

We define the generation set $J_{o}\left(i\right)$ as

(68) $$J_{o}\left(i\right)={\left\{0,\ldots ,G\right\}}^{|T_{o}\left(i-1\right)|-1}\times \left\{0,\ldots ,n\left(i;o\right)-1\right\}.$$

Each vector j in the set corresponds to a possible sequence of past events that led to this point. We define the number of queued packets at the *i*-th allocated slot for the generation vector j for a given starting queue $q_{0}$, denoted as $q_{i}\left(j;q_{0}\right)$, as

(69) $$q_{i}\left(j;q_{0}\right)={\left[q_{i-1}-1\right]}^{+}+j_{i}.$$

If we condition the set on the fact that the packet generated in slot *i* finds *q* packets in the queue, we get

(70) $$p_{\mathrm{gen}}\left(j;o,i,q,q_{0}\right)=\delta \left(q_{\left|j\right|}\left(j;q_{0}\right)-q\right)\mathrm{Bin}\left(j_{\left|j\right|};n\left(i;o\right),\alpha \right)\prod_{k=1}^{|j|-1}\mathrm{Bin}\left(j_{k};G,\alpha \right).$$

For each initial queue $q_{0}$, we can then define a set $C_{o,q_{0}}^{\left(i\right)}\left(l\right)$, which contains the generation vectors that cause exactly *l* transmission opportunities to be unused.

(71) $$C_{o,q_{0}}^{\left(i\right)}\left(l\right)=\left\{j\in J_{o}\left(i\right):\sum_{k=1}^{|j|-1}\delta \left(q_{k}\left(j;q_{0}\right)\right)=l\right\}.$$

We then get $p_{C}\left(l;i,q,o\right)$.

(72) $$p_{C}\left(l;i,q,o\right)=\sum_{q_{0}=0}^{Q}\pi_{q_{0}}^{\left(0\right)}\sum_{j\in C_{o,q_{0}}^{\left(i\right)}\left(l\right)}p_{\mathrm{gen}}\left(j;o,i,q,q_{0}\right).$$

We now repeat the same consideration for transmission opportunities after the transmission of the considered packet. The number of packets in the queue after the transmission V(g), for a given generation set g, is

(73) $$V\left(g\right)=\min\left(\sum_{i=1}^{\left|g\right|}g_{i},Q-1\right).$$

There are at least V(g) occupied transmission opportunities after the transmission of the packets. We can then define the generation set $H_{\ell }\left(V\right)$, which represents the possible new packet arrivals.

(74) $$H_{\ell }\left(V\left(g\right)\right)=\left\{0,\ldots ,\left(V\left(g\right)+1\right)G\right\}\times {\left\{0,\ldots ,G\right\}}^{\ell -1}.$$

The probability of each vector h in the set is given by:

(75) $$p_{\mathrm{gen}}\left(h;\ell ,V\left(g\right)\right)=\mathrm{Bin}\left(h_{1};\left(V+1\right)G,\alpha \right)\prod_{i=2}^{\ell }\mathrm{Bin}\left(h_{i};G,\alpha \right).$$

We define the number of queued packets at the *i*-th allocated slot for the generation vector h, denoted by $q_{i}\left(h\right)$, as

(76) $$q_{i}\left(h\right)={\left[q_{i-1}-1\right]}^{+}+h_{i},$$

where $q_{0}=0$. We can then define the set $F_{\ell }\left(f\right)$, which requires *f* transmission opportunities to be unused by the considered user.

(77) $$F_{\ell }\left(f\right)=\left\{h\in H_{\ell }\left(V\right):\delta \left(f-\sum_{i=1}^{\ell }\delta \left(q_{i}\left(h\right)\right)\right)\right\}.$$

The probability of having *f* unused transmission opportunities for the user by the *d*-th slot in the frame after the packet transmission, given the generation vector g, is then

(78) $$p_{F}\left(f;d,g,o,i,w,q\right)=\sum_{h\in F_{|T_{o}\left(d\right)|-|T_{o}\left(w+i\right)|}\left(f\right)}p_{\mathrm{gen}}\left(h;\ell ,V\left(g\right)\right).$$

In the following, we denote $\left(1-\varepsilon_{B}\right)$ as $p_{1}$ to simplify the notation. We now compute the probability that *r* packets from the broadband user frame are correctly received by slot *d*, given that there are *f* transmission opportunities before it left unused by the considered user:

(79) $$p_{R}\left(r;d,f,o\right)=\sum_{n=0}^{f}\mathrm{Bin}\left(n;f,p_{1}p_{\mathrm{free}}\right)\mathrm{Bin}\left(r-n;d-|T_{o}\left(d\right)|,p_{1}{\left(\pi_{0}^{\left(G-1\right)}\right)}^{\frac{U_{I}}{G}}\right),$$

where $p_{\mathrm{free}}$ is the same as in (29). We can then define the probability that at least *K* packets have been received by slot *d*, $P_{R}\left(d,f,o\right)$.

(80) $$P_{R}\left(d,f,o\right)=\sum_{r=K}^{d}p_{R}\left(r,d;f,o\right).$$

The success probability for a packet *i*, which finds *q* packets ahead of it in the queue, in a frame with offset *o*, is

(81) $$\begin{array}{c} \;\;\;\;\;\;p_{s,I}\left(o,i,q\right)=p_{\mathrm{free}}\left(1-\varepsilon_{I}\right)(\sum_{w=0}^{N-i}[\sum_{l=0}^{|T_{o}\left(i-1\right)|}p_{C}\left(l;i,q,o\right)\sum_{g\in H_{\left\lfloor \frac{w}{G}\right\rfloor }^{\left(n(i;o),q\right)}}p_{\mathrm{gen}}\left(g;\left\lfloor \frac{w}{G}\right\rfloor ,n\right)\\ \times \sum_{f=0}^{|T_{o}\left(N\right)|-|T_{o}\left(w+i\right)|}P_{R}\left(N,f+l,o\right)p_{F}\left(f;N,g,o,i,w,q\right)]+\sum_{w=N-i+1}^{Gq}[\;\;\;\sum_{g\in H_{\left\lfloor \frac{w}{G}\right\rfloor }^{\left(n(i;o),q\right)}}p_{\mathrm{gen}}\left(g;\left\lfloor \frac{w}{G}\right\rfloor ,n\right)\\ \;\;\;\;\;\times \sum_{f=0}^{\left|T_{o}\left(N\left(\left\lfloor \frac{i+w}{N}\right\rfloor +1\right)\right)\right|-\left|T_{o}\left(w+i\right)\right|}P_{R}\left(N,f,\omega_{\left\lfloor \frac{i+w}{N}\right\rfloor }\left(o\right)\right)p_{F}\left(f;N\left(\left\lfloor \frac{i+w}{N}\right\rfloor +1\right),g,o,i,w,q\right)]). \end{array}$$

The latency when the decoding delay is 0:

(82) $$p_{T,Y}\left(t,0;o,i,q\right)=\left\{\begin{array}{cc} \sum_{l=0}^{|T_{o}\left(i-1\right)|}\frac{p_{W}\left(t;n\left(i;o\right),q\right)p_{C}\left(l;i,q,o\right)P_{R}\left(t+i,l,o\right)p_{\mathrm{free}}\left(1-\varepsilon_{I}\right)}{p_{s}^{\left(I\right)}\left(o,i,q\right)} & t+i\leq N;\\ \frac{p_{W}\left(t;n\left(i;o\right),q\right)P_{R}\left(t+i-N\left\lfloor \frac{t+i}{N}\right\rfloor ,0,o\right)p_{\mathrm{free}}\left(1-\varepsilon_{I}\right)}{p_{s}^{\left(I\right)}\left(o,i,q\right)} & t+i>N. \end{array}\right.$$

where $p_{\mathrm{free}}$ is the same as in (29). If the decoding delay is not 0, we need to consider that transmission opportunities after the slot might be free. Furthermore, we define $p_{B}\left(d,i,w,q,g,f,o\right)$ as the probability of correctly receiving a packet from the broadband user in slot *d*:

(83) $$p_{B}\left(d,i,w,q,g,f,o\right)=\left\{\begin{array}{cc} p_{1}{\left(\pi_{0}^{\left(G-1\right)}\right)}^{\frac{U_{I}}{G}} & \mathrm{if}\;d\notin T_{o}\left(d\right);\\ p_{1}p_{\mathrm{free}} & \mathrm{if}\;d\in T_{o}\left(d\right),V\left(g\right)<|T_{o}\left(d\right)|-|T_{o}\left(i+w\right)|;\\ 0 & \mathrm{if}\;d\notin T_{o}\left(d\right),V\left(g\right)\geq |T_{o}\left(d\right)|-|T_{o}\left(i+w\right)|. \end{array}\right.$$

We can now compute the latency and decoding delay joint pmf when the latter is not 0.

(84) $$\begin{array}{c} p_{T,Y}\left(t,t-w;o,i,q\right)=\sum_{g\in H_{\left\lfloor \frac{w}{G}\right\rfloor }^{\left(n(i;o),q\right)}}p_{\mathrm{gen}}\left(g;\left\lfloor \frac{w}{G}\right\rfloor ,n\right)\sum_{l=0}^{|T_{o}\left(i-1\right)|}p_{C}\left(l;i,q,o\right)\sum_{f=0}^{|T_{o}\left(t+i\right)|-|T_{o}\left(w+i\right)|}p_{F}\left(f;i+t,g,o,i,w,q\right)\\ \;\;\;\;\frac{p_{1}\left(1-\varepsilon_{I}\right)}{p_{s,I}\left(o,i,q\right)}p_{\mathrm{free}}p_{B}\left(i+t,i,w,q,g,f,o\right)p_{R}\left(K-1;i+t,f+l,o\right),i+w\leq N. \end{array}$$

If i+w is larger than *N*, the packet is transmitted in the next frame, and we have

(85) $$\begin{array}{c} p_{T,Y}\left(t,t-w;o,i,q\right)=\sum_{g\in H_{\left\lfloor \frac{w}{G}\right\rfloor }^{\left(n,q\right)}}p_{\mathrm{gen}}\left(g;\left\lfloor \frac{w}{G}\right\rfloor ,n\right)\sum_{f=0}^{|T_{o}\left(t+i\right)|-|T_{o}\left(w+i\right)|}p_{F}\left(f;i+t,g,o,i,w,q\right)\frac{p_{1}\left(1-\varepsilon_{I}\right)}{p_{s,I}\left(o,i,q\right)}\\ \;\times p_{\mathrm{free}}p_{B}\left(i+t,i,w,q,g,f,o\right)p_{R}\left(K-1;i+t-N\left\lfloor \frac{i+w}{N}\right\rfloor ,f,o\right),\;i+w>N. \end{array}$$

Now we uncondition $p_{T,Y}\left(t,y;o,i,q\right)$ on *i* and *q* and remove *Y* to get $p_{T}\left(t;o\right)$, knowing that the generation probability is the same in all slots:

(86) $$p_{T}\left(t;o\right)=\sum_{i=1}^{N}\frac{1}{N}\sum_{q=0}^{Q}\pi_{q}^{\left(n(i;o)\right)}\sum_{y=0}^{t}p_{T,Y}\left(t,y;o,i,q\right).$$

Taking the offset set $O(o_{0})$ as defined in (57), and using the probabilities in (58), we get:

(87) $$p_{T}\left(t\right)=\sum_{o\in O(o_{0})}p_{D}\left(o;o_{0}\right)p_{T}\left(t;o\right)\sum_{i=1}^{N}\sum_{q=0}^{Q}\frac{\pi_{q}^{\left(n(i;o)\right)}}{N}p_{s,I}\left(o,i,q\right).$$

In the same way, we can compute the reliability $p_{s}^{\left(I\right)}$ from (81):

(88) $$p_{s,I}=\sum_{o\in O(o_{0})}p_{D}\left(o;o_{0}\right)\sum_{i=1}^{N}\sum_{q=0}^{Q}\frac{\pi_{q}^{\left(n(i;o)\right)}}{N}p_{s,I}\left(o,i,q\right).$$

## 6. Results

**Figure 4:**
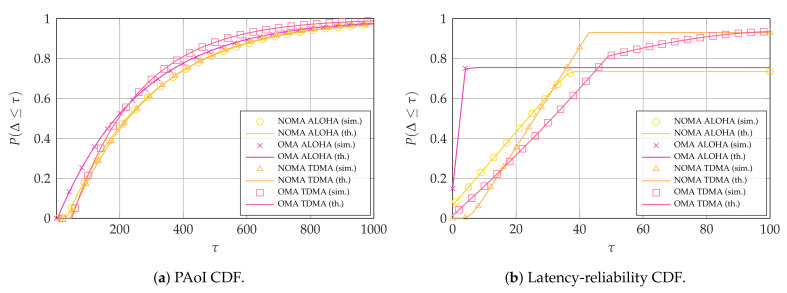
Monte Carlo simulation and theoretical CDFs, with K=32, N=40, and $U_{I}\alpha =0.05$.

In this section, we show some illustrative analytical results for the PAoI-oriented and latency-oriented case. We first confirm that our theoretical calculations are correct by considering a given scenario and performing a Monte Carlo simulation. We simulate the erasure channel and destructive interference simply by dropping packets from the list, and consider T=1,000,000 frames. In the scenario we simulate, the broadband user protects its transmission with a *K* over *N* erasure code, i.e., N=40 and K=32, and the arrival rate for each of the $U_{I}=10$ intermittent users is α=0.005 (i.e., $U_{I}\alpha =0.05$). The OMA systems use $T_{\mathrm{int}}=5$. As Figure 4 shows, the theoretical results for both PAoI and latency-reliability, shown here as CDFs, match the simulations perfectly in all cases. Monte Carlo results are not shown for the rest of the section to improve the understandability of the plots, but the results still match tightly with the theoretical analysis.

The results are presented in the form of Pareto frontiers, that capture the best trade-offs between the throughput of the broadband user $S_{B}$ and the 90th percentile of the timeliness metric for the intermittent users. The parameter settings are shown in Table 2. With the selected parameters and if only the broadband user is considered, the optimal source and coded block sizes are N=77 and K=64, where *K* is limited to 64 to make the solution practical), respectively, which results a throughput of $S_{B}=0.8147$ packets per slot. The latter corresponds to the upper bound in throughput for both OMA and NOMA systems evaluated in the following.

**Figure 5:**
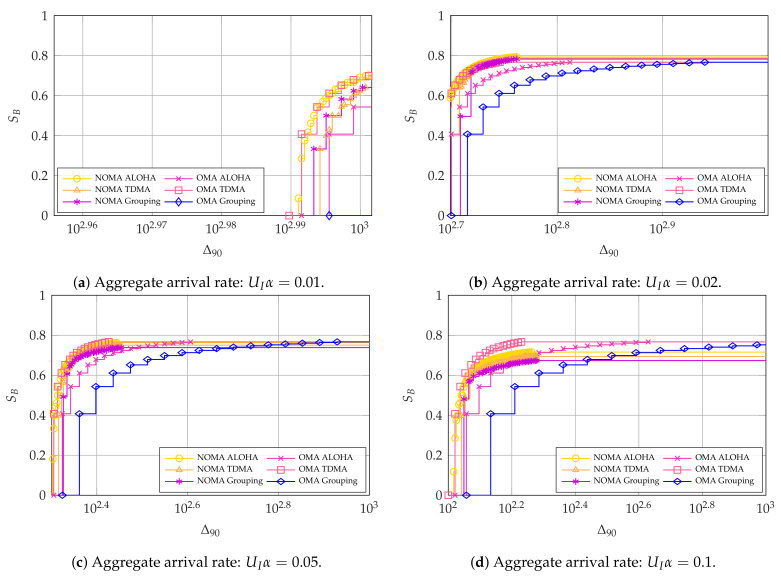
Pareto frontier for $S_{B}$ and $\Delta_{90}$ with $U_{I}=4$. For OMA, $K^{*}=64$ and $N^{*}=77$.

We first consider PAoI-oriented systems, whose performance has a strong dependence on the aggregate arrival rate $U_{I}\alpha$. We assume that $U_{I}=4$, which allows us to explore a wide range of values for α. In this case, the grouping scheme used G=2, whereas the ALOHA and TDMA cases had the expected G=1 and G=4, respectively. When α is very low, the inter-arrival time dominates the PAoI and the impact of the choice of access schemes is negligible. As Figure 5 shows, this is true even for a total arrival rate of $U_{I}\alpha =0.01$, which corresponds to an average of one packet every 400 slots from each source: as the arrival process is exponentially distributed, the 90th percentile of the inter-arrival time is 920 slots, and it is impossible to achieve a lower $\Delta_{90}$. In cases with a higher arrival rate, OMA TDMA seems to be the best system, although NOMA ALOHA can achieve a similar performance when PAoI is more important than the broadband user throughput.

**Figure 6:**
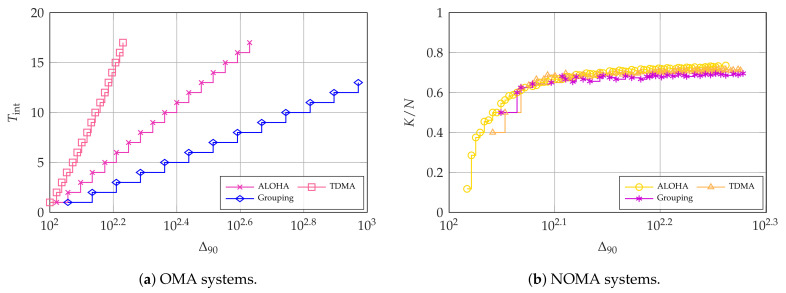
Pareto-optimal configurations for $S_{B}$ and $\Delta_{90}$ with $U_{I}=4$ and α=0.025. For OMA, $K^{*}=64$ and $N^{*}=77$.

Besides the achievable performance trade-offs, it is also important to observe the parameter settings that achieve Pareto efficiency, as shown in Figure 6. The difference between the optimal values of $T_{\mathrm{int}}$ in OMA for the three considered schemes is stark, as shown in Figure 6a. This is because collisions are the main factor driving up the age in OMA, making the age for TDMA far lower. The other factor in the age is the waiting time due to the grouping: while TDMA compensates for this by avoiding collisions entirely, the grouping scheme with G=2 is the worst of both worlds, getting extremely poor performance due to having both a longer interval between allocated slots and the risk of collisions. Therefore, in age-oriented systems where the arrival rate α for each intermittent user needs to be relatively high to achieve the desired AoI, orthogonal slicing among all users (broadband and intermittent) is a good choice, as the alternative will result in a high collision probability.

Collisions are not so common in TDMA, as the transmissions for the intermittent users can be spread out over all slots, and are not concentrated in some reserved ones. In this case, the specific method used is not very important, as Figure 6b shows: the three schemes have a similar age with very similar coding rates. However, NOMA cannot significantly outperform OMA TDMA, as allowing collisions with the broadband user limits the achievable throughput.

**Figure 7:**
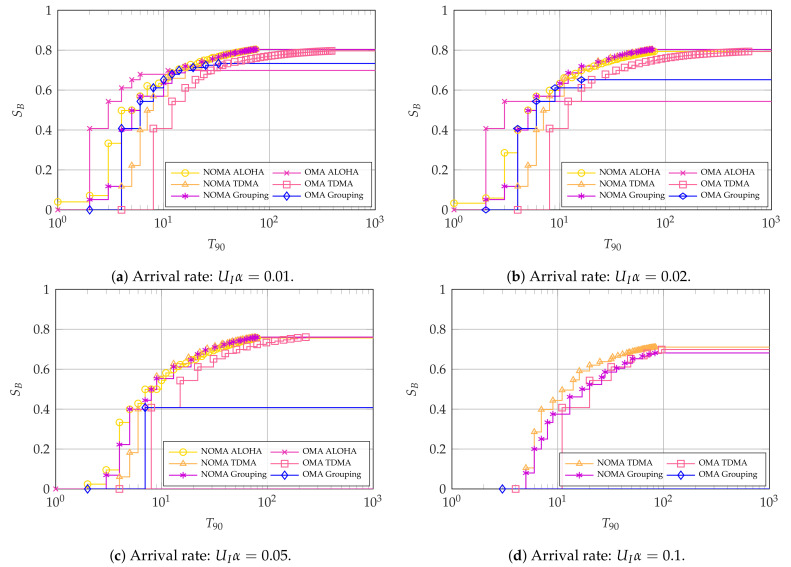
Pareto frontier for $S_{B}$ and $T_{90}$ with $U_{I}=4$. For OMA, $K^{*}=64$ and $N^{*}=77$.

**Figure 8:**
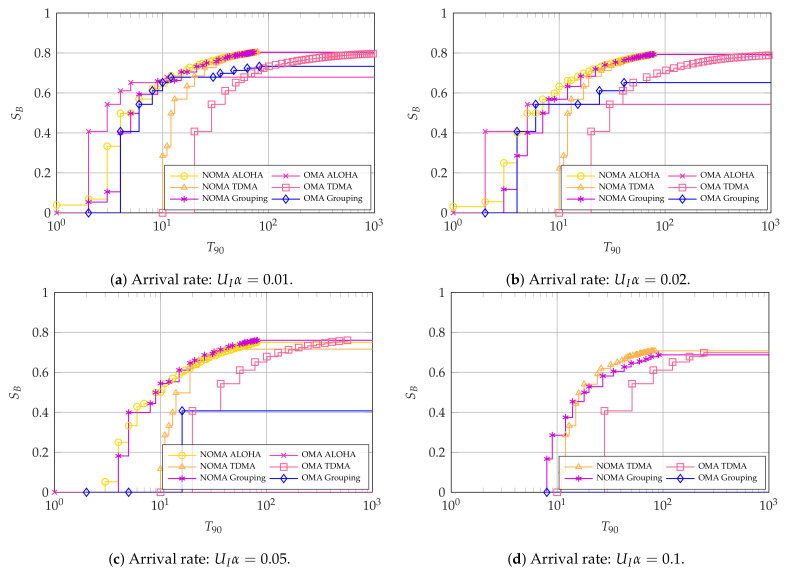
Pareto frontier for $S_{B}$ and $T_{90}$ with $U_{I}=10$. For OMA, $K^{*}=64$ and $N^{*}=77$.

**Figure 9:**
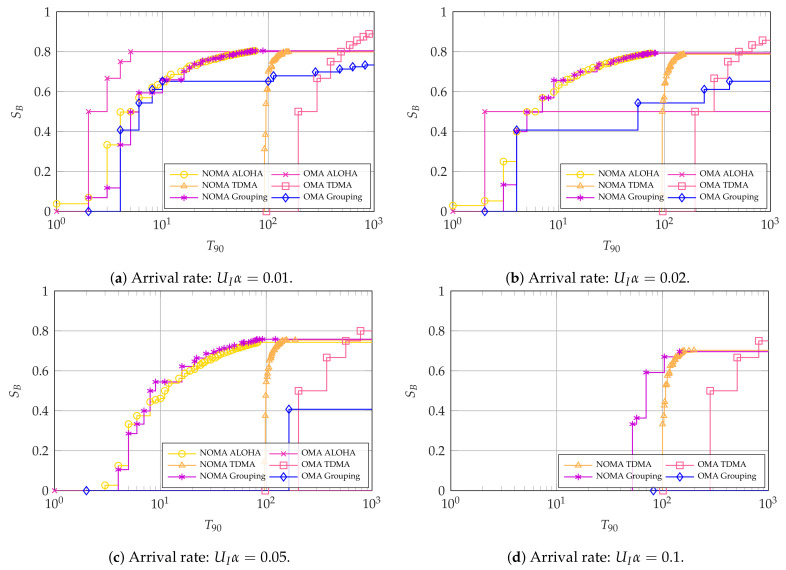
Pareto frontier for $S_{B}$ and $T_{90}$ with $U_{I}=100$. For OMA, $K^{*}=64$ and $N^{*}=77$.

Next, we consider the latency-oriented systems where the 90th percentile of latency-reliability $T_{90}$ is the main KPI for intermittent users. For these, we focus on illustrating the impact of the arrival rate $U_{I}\alpha$ and the number of intermittent users $U_{I}$. Figure 7, Figure 8 and Figure 9 show the Pareto frontiers for the cases with $U_{I}=4$, $U_{I}=10$, and $U_{I}=100$, respectively. Each of the figures includes the latency and throughput trade-offs for $U_{I}\alpha \in \left\{0.01,0.02,0.05,0.1\right\}$.

For the case with $U_{I}=4$, we see an interesting phenomenon in Figure 7a,b: if the arrival rate is low, OMA ALOHA is the optimal choice if the main KPI is the latency-reliability. However, it is not able to achieve a high broadband user throughput $S_{B}$. Conversely, NOMA, either with ALOHA or grouped access among the intermittent users, can achieve the greatest throughput $S_{B}≃0.8$. In addition, NOMA ALOHA achieves the lowest latency-reliability with $S_{B}>0$.

As the arrival rate increases with $U_{I}=4$, NOMA becomes the Pareto efficient choice for all points in the latency-throughput trade-off, albeit with a small margin. This is observed in Figure 7c,d, where the Pareto efficient methods are NOMA ALOHA and NOMA TDMA, respectively, with NOMA grouping achieving a close performance. The reason for the better performance of NOMA with high arrival rates is that it allows the intermittent users to access considerably more resources than OMA, which minimizes collisions between them. These collisions are considerably harmful for the system as they cannot be resolved. Therefore, OMA ALOHA becomes infeasible with high arrival rates, whereas OMA TDMA may suffer from queue overflows since intermittent slots are spaced by $U_{I}T_{\mathrm{int}}$ slots.

Next, Figure 8 shows a similar pattern to Figure 7, but with a much better performance of NOMA with respect to OMA. Specifically, NOMA ALOHA and grouping achieve much better trade-offs when compared to OMA TDMA for the considered arrival rates, with the only exception being that NOMA grouping is not viable for $U_{I}\alpha =0.1$. This is also the case with all the ALOHA methods, which fail for the cases with $U_{I}\alpha =0.1$ because of the excessive collisions among the intermittent users. Finally, OMA grouping can only achieve the required 90% reliability for the intermittent users with $U_{I}\alpha =0.1$ by making $S_{B}=0$.

The case with $U_{I}=100$, displayed in Figure 9, features a more pronounced differences among the access schemes, indicating that the selection of the access scheme and/or its parameters will be even more critical in massive access scenarios with larger number of users. As in the previous cases, using NOMA becomes more convenient as the total arrival rate increases. OMA ALOHA performs particularly well for low total activation rates, as collisions between intermittent users are rare in this scenario, and in settings that are oriented more towards latency-reliability than broadband user throughput, as increasing the transmission opportunities for the intermittent users can further reduce the probability of collisions between them.

In general, it can be concluded that ALOHA schemes perform better under low arrival rates $U_{I}\alpha$, whereas TDMA schemes perform better when the aggregate arrival rate increases. This may be expected, in particular as the assumed timeliness parameters of interest are rather stringent. The performance of OMA grouping oftentimes lies between that of OMA ALOHA and TDMA for all values of $U_{I}$. This showcases its robustness to the arrival rate $U_{I}\alpha$, but also that it is not an ideal option to optimize performance. Instead, NOMA grouping achieves a remarkable performance, oftentimes matching or even surpassing the performance of NOMA ALOHA and NOMA TDMA, even with very high rates. Depending on the scenario, the number of groups is highly variable: if $U_{I}\alpha =0.1$, the grouping scheme uses the largest possible number of groups (i.e., G=50 with $U_{I}=100$), making the scheme closer to TDMA than pure ALOHA. On the other hand, ALOHA is more convenient for lower activation rates, so the best grouping performance will be obtained with G=2.

Most interestingly, NOMA schemes outperform OMA under most conditions, with the exception of OMA ALOHA for low arrival rates. This behavior is extremely encouraging for the performance of NOMA in realistic systems, as the collision channel we considered is a worst-case scenario for non-orthogonal access.

As observed in our previous work [7], by including the probability of channel capture and intra-collision SIC, the performance of non-orthogonal schemes can only improve. Nevertheless, OMA may can also benefit from capture and intra-collision SIC by mitigating collisions between intermittent users.

## 7. Conclusions

In this work, we investigated the performance trade-offs with orthogonal and non-orthogonal spectrum slicing in a multiple access system with broadband and intermittent users. We derived closed-form expressions for both PAoI and latency-reliability for the intermittent users, along with throughput for the broadband user, in a time-slotted system in which the users share a single frequency channel.

The results illustrate that, by implementing an erasure code at the broadband user, the choice between OMA and NOMA depends on the specific features of the considered scenario and on the objectives of the system designer. In particular, the number of intermittent users and their aggregate arrival rate have major impacts on the preferred slicing and access method for latency-oriented systems. In these cases, TDMA was clearly preferable for the higher arrival rates, whereas ALOHA performed remarkably well with low to medium arrival rates. Interestingly, the opposite effect can be seen for the choice of the access scheme, as NOMA outperformed OMA with higher arrival rates, and orthogonal allocation worked better for lower arrival rates. The NOMA ALOHA scheme presents a case of particular interest, as by correctly tuning the coding parameters for the broadband user, it could oftentimes achieve the best performance trade-offs with low to medium arrival rates in the extreme cases—that is, when the intermittent users required the lowest latency and when the broadband users required the highest throughput. On the other hand, NOMA TDMA is clearly the best access method for latency-reliability with high arrival rates. The PAoI results show that the two access methods are almost equivalent, as long as they are configured properly, and the main driver of performance is the packet generation process. However, OMA TDMA does show significant advantages with respect to the other OMA schemes, as it avoids collisions entirely, whereas the other OMA schemes may still have collisions between intermittent users. These results, obtained in the simple collision channel without capture, showcase the potential of NOMA schemes in scenarios with heterogeneous service types as channel capture and intra-collision SIC greatly improve its performance.

Future work on the subject can be oriented in multiple directions: First, analyzing the system with MPR is definitely a priority, as the worst-case analysis has already shown the advantages of NOMA. Secondly, more realistic systems could be investigated, with time-dependent arrival patterns or with multiple frequency channels, which would add an interesting dimension to the problem by providing parallel resources. The possibility of using packet repetition to increase the intermittent users’ reliability is another interesting facet that can be examined, although the complexity of the system may grow beyond the possibility of analytical tools, requiring a simulation-based approach.

## Figures and Tables

**Table 1:** Main notation used in the paper.

Symbol	Meaning	Symbol	Meaning
U	Set of users	*U*	Cardinality of U\{1}
*K*	Information packets in a frame	*N*	Total packets in a frame
$A_{t}$	Set of users that can transmit in *t*	$a_{u,t}$	Indicator of user *u*’s activity in *t*
$\varepsilon_{B}$	Broadband user channel error	$\varepsilon_{I}$	Intermittent user channel error
Δ	Peak Age of Information (PAoI)	*T*	Latency-reliability
$\Delta_{90}$	90th perc. of PAoI	$T_{90}$	90th perc. of latency-reliability
Mult(k;N,p)	Multinomial function	Bin(k;N,p)	Binomial function
*G*	Number of groups	α	Intermittent user activation rate
$S_{B}$	Broadband user throughput	$p_{s,B}$	Frame decoding probability
$T_{\mathrm{int}}$	OMA intermittent slot period	ρ	Intermittent slot activation rate
$p_{s,I}$	Intermittent user success prob.	*W*	Waiting delay
*Z*	Inter-transmission time	*Q*	Intermittent user queue size
$P^{\left(Q\right)}$	Queue state transition matrix	$\pi^{\left(n\right)}$	*n*-th slot steady-state distribution
$G_{\ell }^{\left(n\right)}$	*n*-th slot generation set	$p_{\mathrm{gen}}\left(g;\ell ,n\right)$	*n*-th slot generation probability
$\psi_{\ell }^{g,q}$	Transmission condition for g	$H_{\ell }^{\left(n,q\right)}$	Valid transmission vector set
$T_{o}\left(d\right)$	NOMA transmission slot set	$p_{\mathrm{tx}}\left(m;d,o\right)$	Prob. of transmitting *m* packets
*F*	First intermittent decoding	*⌀*	No int. decoding in a frame
*R*	Int. decoding in a given slot	*E*	A given int. decoding is the first
*L*	A given int. decoding is the last	ω	Offset of the next frame
*M*	Consecutive empty frames	O	Set of possible offsets
*O*	Decoding with a given offset	*D*	A packet is dropped
$J_{o}\left(i\right)$	Past generation set	$C_{o,q_{0}}^{\left(j\right)}\left(i\right)$	Future generation set
*C*	Unused future slots	*V*	Number of packets left in queue
$H_{\ell }\left(V\left(g\right)\right)$	Future arrival set	$F_{\ell }\left(f\right)$	Set of unused past slots
*Y*	Decoding delay	$p_{\mathrm{free}}$	Free channel probability

**Table 2:** System parameters.

Parameter	Symbol	Setting
Source block size for broadband user	*K*	{1,2,⋯,64}
Coded block size	*N*	≥K
Erasure probability for the broadband user	$\varepsilon_{B}$	0.1
Erasure probability for intermittent users	$\varepsilon_{I}$	0.05
Total intermittent arrival rate [packets per slot]	$U_{I}\alpha$	{0.01,0.02,0.05,0.1}
Number of intermittent users	$U_{I}$	{4,10,100}
OMA: Period between intermittent slots	$T_{\mathrm{int}}$	{1,2,⋯,64}
Maximum queue length	*Q*	{1,4}

## Data Availability

Not applicable.
